# Breast Cancer Prevalence and Mortality among Hispanic Subgroups in the United States, 2009–2013

**DOI:** 10.1155/2016/8784040

**Published:** 2016-09-08

**Authors:** Bijou R. Hunt

**Affiliations:** Sinai Urban Health Institute, Sinai Health System, 1500 South Fairfield Avenue, Chicago, IL 60608-1797, USA

## Abstract

*Background*. This paper presents data on breast cancer prevalence and mortality among US Hispanics and Hispanic subgroups, including Cuban, Mexican, Puerto Rican, Central American, and South American.* Methods*. Five-year average annual female breast cancer prevalence and mortality rates for 2009–2013 were examined using data from the National Health Interview Survey (prevalence) and the National Center for Health Statistics and the American Community Survey (mortality rates).* Results*. Overall breast cancer prevalence among US Hispanic women was 1.03%. Although the estimates varied slightly by Hispanic subgroup, these differences were not statistically significant. The breast cancer mortality rate for Hispanics overall was 17.71 per 100,000 women. Higher rates were observed among Cubans (17.89), Mexicans (18.78), and Puerto Ricans (19.04), and a lower rate was observed among Central and South Americans (10.15). With the exception of the rate for Cubans, all Hispanic subgroup rates were statistically significantly different from the overall Hispanic rate. Additionally, all Hispanic subgroups rates were statistically significantly higher than the Central and South American rate.* Conclusion*. The data reveal significant differences in mortality across Hispanic subgroups. These data enable public health officials to develop targeted interventions to help lower breast cancer mortality among the highest risk populations.

## 1. Introduction

The consistent growth of the Hispanic population in the United States (US) over the past two decades [1, 2] has prompted the need for disaggregated data on Hispanic subgroups. As a result, a growing body of research has emerged which examines differences in the morbidity and mortality experiences of various Hispanic subgroups in the US. This literature reveals large differences among subgroups on a variety of health outcomes and behaviors (e.g., all-cause mortality [3–6], cancer [7–11], and other cause-specific mortalities [9, 12–14]; diet [15], smoking [16, 17], and alcohol use [16]) and generally concludes that the Hispanic label masks important heterogeneity and cautions against extrapolating data from one Hispanic subgroup to another [3, 7, 12, 18].

Despite this recent proliferation of research examining health outcomes among Hispanic subgroups, one topic on which there continues to be a lack of data is breast cancer. Breast cancer is the most commonly diagnosed cancer in Hispanic women, as well as the leading cause of cancer death for this group [19]. Given recently documented differences in the prevalence of and mortality from cancer across Hispanic subgroups [12], it is reasonable to expect that similar differences might exist for breast cancer.

Research has shown that breast cancer outcomes are more favorable when the disease is detected in its early stages and is thus amenable to early intervention [12, 20, 21]. However, without prevalence and mortality data to inform which groups are most affected, it is not possible to target programmatic and policy interventions to those with the greatest need [7, 11, 22]. The present analysis thus aims to provide data on breast cancer prevalence and mortality among Hispanics and Hispanic subgroups in the US. Five-year average annual age-adjusted female breast cancer prevalence and mortality rates for 2009–2013 are examined. Sociodemographic variables for 2013 are also shown. Data are presented for the US population, US-born, foreign-born, non-Hispanic Whites, Hispanics, and Hispanic subgroups, including Cuban, Mexican, Puerto Rican, Central American, and South American. These data will allow public health officials to develop targeted interventions to help lower breast cancer mortality among the highest risk populations.

## 2. Materials and Methods

### 2.1. Sociodemographic Variables

Selected sociodemographic variables were examined using self-reported data from the 2013 American Community Survey (ACS) 1-year estimates [23]. ACS variables include population size, the percent of the Hispanic population comprised by each Hispanic subgroup, the percent of the population aged 18–64, the percent of the population over 25 years that is a high school graduate or higher, and the percent of the population over 16 years that is unemployed.

### 2.2. Age-Adjusted Breast Cancer Prevalence Estimates

Data from the National Health Interview Survey (NHIS) for the period 2009–2013 were used to calculate the prevalence of female breast cancer among adults aged 18–64 for the US population, non-Hispanic Whites, Hispanics, and Hispanic subgroups, including Cuban/Cuban American, Mexican, Mexican American, Puerto Rican, and Central or South American. Data for Mexican and Mexican American subgroups were pooled into a single category, Mexican American. Data were stratified by nativity (US-born versus foreign-born).

### 2.3. Age-Adjusted Breast Cancer Mortality Rates

Age-adjusted female breast cancer mortality rates were calculated for the US population, non-Hispanic Whites, Hispanics, and Hispanic origin subgroups, including Cuban, Mexican, Puerto Rican, Central American, and South American. Data for Central American and South American subgroups were pooled into a single category, Central/South American [12]. Numerator data for 2009–2013 were abstracted from death files obtained from the National Center for Health Statistics [24]. Deaths where the cause was malignant neoplasm of the breast (ICD-10 = C50) for women were included in this analysis. Population-based denominators were obtained from the US Census Bureau, 2006–2010 American Community Survey. The racial/ethnic-specific and sex-specific classification ratios derived by the CDC were applied to correct for misreporting of race/ethnicity on death certificates [12, 25].

For each of the data sources—the death files, the Census, and the NHIS—race/ethnic classifications were obtained by cross-tabulating two variables: Hispanic ethnicity and racial identity.

For the Census and the NHIS, reporting of race and ethnicity is based on respondent self-report. Hispanic origin is determined by self-reported country of origin and includes differing options for the Census (Mexican, Mexican American, or Chicano; Puerto Rican; Cuban; and another Hispanic, Latino, or Spanish origin with an option to write in the origin country) and the NHIS (Mexican, Puerto Rican, Cuban, South or Central American, or another Spanish culture or origin).

For the death certificate, reporting of race and ethnicity is typically the responsibility of a funeral director who gathers this information from next of kin or relies on personal observation [25]. Mortality data for decedents of Central American or South American decent were pooled into a single “Central/South American” category as some states reported their data in this fashion [12].

Age-adjusted prevalence (expressed as a percentage) and mortality rates (per 100,000 population) were calculated employing the 2000 standard US population. For each estimate and rate, we calculated 95% confidence intervals (CIs) [26] and considered nonoverlapping CIs to indicate a statistically significant difference [12]. All data were analyzed using SAS 9.3.

## 3. Results

Table 1 displays sociodemographic characteristics of the US female population by nativity, race/ethnicity, and Hispanic subgroup. Mexicans make up the largest proportion (63.4%) of the Hispanic female population in the US, followed by Puerto Ricans (9.8%), Central Americans (8.6%), South Americans (6.4%), and Cubans (3.8%). The percent of the population aged 18–64 is around 60% for Mexicans, Puerto Ricans, and Cubans and closer to 65% for Central and South Americans. Central Americans (57.1%) display the lowest percentage of high school graduates, while South Americans display the highest percentage (85.3%). The unemployment rate is the highest among Puerto Ricans (7.4%), despite their relatively high percentage of high school graduates (78.2%).

Table 2 displays the 2009–2013 age-adjusted breast cancer prevalence estimates for the US and by race and ethnicity. Overall, 1.25% of women in the US have been diagnosed with breast cancer, with US-born women displaying a slightly higher estimate than their foreign-born counterparts (1.28% versus 1.07%, resp.; differences are not statistically significant). White women experience a higher prevalence (1.32%) compared to the US average and, within this group, the estimate is lower for US-born compared to foreign-born women (1.30% versus 1.60%, resp.; differences are not statistically significant).

The overall breast cancer prevalence among US Hispanic women is 1.03%. Although the estimates vary slightly by Hispanic subgroup, these differences are not statistically significant. Similarly, while the prevalence of breast cancer is higher among US-born Mexican women (1.18%) than their foreign-born counterparts (0.83%), the differences are not statistically significant. Similar comparisons could not be made for other Hispanic subgroups. For Cubans the relative standard errors were greater than 30%. For Puerto Ricans, the foreign-born population did not report any breast cancer diagnoses. Similarly, for Central/South Americans, the US-born population did not report any breast cancer diagnoses.

Table 3 displays the 2009–2013 age-adjusted breast cancer mortality rates for the US, by race and ethnicity. The mortality rate for the total female US population is 22.73 per 100,000 women. Among the groups examined in the present analysis, the rate is the highest among non-Hispanic White women at 22.49. The mortality rate for Hispanics overall is 17.71 per 100,000 women. Higher rates are observed among Cubans (17.89), Mexicans (18.78), and Puerto Ricans (19.04), and a lower rate is observed among Central and South Americans (10.15). With the exception of the rate for Cubans, all Hispanic subgroup rates are statistically significantly different from the overall Hispanic rate. Additionally, all Hispanic subgroups rates are statistically significantly higher than the Central and South American rate.

## 4. Discussion

The present analysis revealed significant differences in breast cancer mortality rates among Hispanic subgroups in the US. Not only do differences exist between the subgroups which make up the Hispanic population, but also differences exist between the overall Hispanic estimate and the specific subgroups. In particular, Mexican and Puerto Rican women have a higher breast cancer mortality rate than the total Hispanic female population and compared to Central and South American women. Interestingly, no significant differences were found in the prevalence of breast cancer among the Hispanic subgroups. These disaggregated estimates of prevalence and mortality are critical for informing targeted cancer control and prevention efforts [7].

A recent report by the CDC provides detailed data on overall cancer prevalence and mortality by Hispanic ethnicity and subgroup for the same years as the present study, 2009–2013 [12]. The authors found that the prevalence of cancer varied among Hispanic subgroups, with Mexicans displaying a profile most similar to the overall Hispanic group and Puerto Ricans displaying a much higher estimate than those for Hispanics, Mexicans, and Central/South Americans. While similar patterns were observed in the present findings related to breast cancer, there were not statistically significant differences in prevalence across these groups. With regard to the overall cancer mortality findings from the CDC report, the rates for Puerto Ricans and Cubans (but not Mexicans) were statistically significantly different from the rate for Hispanics. For breast cancer, all Hispanic subgroup rates, with the exception of the rate for Cubans, were statistically significantly different from the rate for Hispanics overall. Additionally, all Hispanic subgroups rates were statistically significantly higher than the Central and South American rate.

While our findings reflect lower mortality rates for Hispanics compared to non-Hispanic Whites, this comparison should not undermine the large contribution of breast cancer to morbidity and mortality among Hispanics. Indeed, as has been noted previously, “even if Hispanics have lower rates of the most common cancers than non-Hispanics, such sites are still the most important cancers among Hispanic populations” [11]. Furthermore, despite more favorable outcomes regarding breast cancer prevalence and mortality, compared to non-Hispanic White women, Hispanic women display lower rates of mammography use [12], have experienced a slower rate of decline in incidence rates since 2000, are more likely to die from their breast cancer, are diagnosed at younger ages, and have higher rates of high-grade and estrogen receptor negative tumors [27]. There is also evidence that acculturation impacts the breast cancer reproductive and hormonal risk profile, with more assimilated women having a higher likelihood of early menarche and a lower likelihood of a late first birth or ever breastfeeding [28].

### 4.1. Methodological Considerations, Strengths, and Limitations

It is possible that disease prevalence may be underestimated among groups with lower insurance coverage and/or access to health care [12]. To the extent that this is the case among the Hispanic population, this could impact knowledge of disease and drive down estimates of breast cancer prevalence. Given Hispanics' lower rates of mammography usage compared to Whites [12], this is not an unreasonable scenario. Research has shown that Hispanic ethnicity/origin is underreported on death certificates, but the present study used the ethnic- and sex-specific classification ratios derived by the National Center for Health Statistics from the National Longitudinal Mortality Study [25]. The fact that country of origin data are aggregated to “Central” and “South” American and that these populations are relatively small in the US means that we are unable to explore possible heterogeneity among persons from countries in these geographical areas. The main strength of this analysis is that, through the use of multiple national data sources and multiple years of data, we are able to present data on Hispanic subgroups that are not often accessible.

The Hispanic population is young, heterogeneous, and growing, which means that understanding and addressing this group's diverse health needs is of great importance to the overall health of the nation. Previous research has suggested a link between poorer breast cancer survival outcomes and socioeconomic indicators like inadequate health insurance coverage [29]. Given the recent passage of the Affordable Care Act (ACA), the proportion of uninsured persons should be declining, making this an optimal time to develop culturally and linguistically appropriate interventions that can be targeted to specific Hispanic subgroups. The data presented in this analysis can help guide these efforts.

## Figures and Tables

**Table 1:** Selected sociodemographic characteristics of the US female population, by nativity, race/ethnicity, and Hispanic subpopulation: American Community Survey, 2013.

Characteristic	Population	% of Hispanic population	% population aged 18–64	% high school graduate or higher^a^	% unemployed^b^
US population	160,501,141		62.0	87.2	4.8
US-born	139,313,852		—	—	5.4
Foreign-born	21,170,210		—	—	5.0
White, non-Hispanic	100,164,054		61.8	92.1	3.7
Hispanic	26,577,169	100.0	60.3	66.1	6.5
Hispanic subpopulation					
Mexican	16,843,425	63.4	58.6	60.5	6.4
Puerto Rican	2,599,883	9.8	61.1	78.2	7.4
Cuban	1,014,630	3.8	61.3	79.3	5.7
Central American	2,285,947	8.6	64.8	57.1	6.8
South American	1,691,956	6.4	67.8	85.3	5.6

*Source*: US Census Bureau, American FactFinder, 2013, 1-year estimates.

^a^Among those aged ≥25 years.

^b^Among those aged ≥16 years.

**Table 2:** Breast cancer prevalence estimates by race and ethnicity, United States: 2009–2013.

Population group	Prevalence estimate (%)^a^	95% CI
US population	1.25	1.14–1.35
US-born	1.28	1.16–1.39
Foreign-born	1.07	0.87–1.27
Non-Hispanic White	1.32	1.19–1.45
US-born	1.30	1.17–1.43
Foreign-born	1.60	0.94–2.26
Hispanic	1.03	0.81–1.25
US-born	1.20	0.82–1.58
Foreign-born	0.93	0.65–1.22
Cuban	—^b^	—^b^
US-born	—^b^	—^b^
Foreign-born	—^b^	—^b^
Mexican	0.92	0.67–1.18
US-born	1.18	0.72–1.63
Foreign-born	0.83	0.41–1.25
Puerto Rican	1.53	0.62–2.44
US-born	1.55	0.63–2.48
Foreign-born	0.0	0.0–0.0
Central and South American	0.94	0.46–1.42
US-born	0.0	0.0–0.0
Foreign-born	1.07	0.50–1.63

*Source*: National Health Interview Survey.

CI = confidence interval.

^a^Breast cancer prevalence estimate is age-adjusted using the US 2000 Standard Population for ages 18–64 years using age groups 18–44, 45–54, and 55–64. Breast cancer is based on self-reported responses to questions about (1) whether respondents had ever been told by a doctor or other health professional that they had breast cancer or a malignancy of any kind and (2) what kind of cancer it was (breast). Unknowns for the column were not included when calculating percentages.

^b^Estimate has a relative standard error >30%.

**Table 3:** Breast cancer mortality rates by race and ethnicity, United States: 2009–2013.

Population group	Mortality rate^a^	95% CI
US population	22.73	22.63–22.83
Non-Hispanic White	22.49	22.37–22.60
Hispanic	17.71	17.41–18.02
Cuban	17.89	16.79–19.00
Mexican	18.78	18.34–19.22
Puerto Rican	19.04	18.08–19.99
Central and South American	10.15	9.53–10.77

*Source*: National Center for Health Statistics.

CI = confidence interval.

^a^Age-adjusted breast cancer mortality rate is expressed per 100,000 females using the US 2000 Standard Population.

## References

[1] Ennis S. R., Ríos-Vargas M., Albert N. G. http://www.census.gov/prod/cen2010/briefs/c2010br-04.pdf.

[2] U.S. Hispanic/Latino Population http://www.infoplease.com/ipa/A0779064.html.

[3] Borrell L. N., Crawford N. D. (2009). All-cause mortality among Hispanics in the United States: exploring heterogeneity by nativity status, country of origin, and race in the National Health Interview Survey-linked Mortality Files. *Annals of Epidemiology*.

[4] Sorlie P. D., Backlund E., Johnson N. J., Rogot E. (1993). Mortality by Hispanic status in the United States. *The Journal of the American Medical Association*.

[5] Palloni A., Arias E. (2004). Paradox lost: explaining the Hispanic adult mortality advantage. *Demography*.

[6] Borrell L. N., Lancet E. A. (2012). Race/ethnicity and all-cause mortality in US adults: revisiting the Hispanic paradox. *American Journal of Public Health*.

[7] Martinez-Tyson D., Pathak E. B., Soler-Vila H., Flores A. M. (2009). Looking under the Hispanic umbrella: cancer mortality among Cubans, Mexicans, Puerto Ricans and other Hispanics in Florida. *Journal of Immigrant and Minority Health*.

[8] Mallin K., Anderson K. (1988). Cancer mortality in Illinois Mexican and Puerto Rican immigrants, 1979–1984. *International Journal of Cancer*.

[9] Rosenwaike I. (1987). Mortality differentials among persons born in Cuba, Mexico, and Puerto Rico residing in the United States, 1979–81. *American Journal of Public Health*.

[10] Rosenwaike I. (1984). Cancer mortality among Puerto Rican-born residents in New York City. *American Journal of Epidemiology*.

[11] Trapido E. J., Burciaga Valdez R., Obeso J. L., Strickman-Stein N., Rotger A., Pérez-Stable E. J. (1995). Epidemiology of cancer among Hispanics in the United States. *Journal of the National Cancer Institute. Monographs*.

[12] Dominguez K., Penman-Aguilar A., Chang M. H. (2009). Vital signs: leading causes of death, prevalence of diseases and risk factors, and use of health services among Hispanics in the United States—2009–2013. *Morbidity and Mortality Weekly Report*.

[13] Hummer R. A., Rogers R. G., Amir S. H., Forbes D., Frisbie W. P. (2000). Adult mortality differentials among Hispanic subgroups and non-Hispanic whites. *Social Science Quarterly*.

[14] Rosenwaike I., Rosenwaike I. (1991). Mortality experience of hispanic populations. *Mortality of Hispanic Populations: Mexicans, Puerto Ricans, and Cubans in the United States and in the Home Countries*.

[15] Loria C. M., Bush T. L., Carroll M. D. (1995). Macronutrient intakes among adult Hispanics: a comparison of Mexican Americans, Cuban Americans, and mainland Puerto Ricans. *American Journal of Public Health*.

[16] NAS (2006). *Hispanics and the Future of America*.

[17] Hunt B. R., Whitman S. (2011). Maternal smoking in chicago: a community-level analysis. *Journal of Health Care for the Poor and Underserved*.

[18] Modiano M. R., Villar-Werstler P., Meister J., Figueroa-Vallés N. (1995). Cancer in Hispanics: issues of concern. *Journal of the National Cancer Institute. Monographs*.

[19] American Cancer Society (2015). *Cancer Facts & Figures for Hispanics/Latinos 2015–2017*.

[20] Caplan L. (2014). Delay in breast cancer: implications for stage at diagnosis and survival. *Frontiers in Public Health*.

[21] Tabar L., Yen M.-F., Vitak B., Chen H.-H. T., Smith R. A., Duffy S. W. (2003). Mammography service screening and mortality in breast cancer patients: 20-year follow-up before and after introduction of screening. *The Lancet*.

[22] Ramirez A. G. M., McAlister A. L., Villarreal R. (1998). Prevention and control in diverse Hispanic populations: a national initiative for research and action. *Cancer*.

[23] US Census Bureau (2013). *American Community Survey*.

[24] National Center for Health Statistics

[25] Arias E., Schauman W. S., Eschbach K., Sorlie P. D., Backlund E. (2008). The validity of race and Hispanic origin reporting on death certificates in the United States. *Vital and Health Statistics, Series 2: Data Evaluation and Methods Research*.

[26] Kleinbaum D. G., Kupper L. L., Morgenstern H. (1982). *Epidemiologic Research: Principles and Quantitative Methods*.

[27] Haile R. W., John E. M., Levine A. J. (2012). A review of cancer in U.S. Hispanic populations. *Cancer Prevention Research*.

[28] Nodora J. N., Gallo L., Cooper R. (2014). Reproductive and hormonal risk profile according to language acculturation and country of residence in the ella binational breast cancer study. *Journal of Women's Health*.

[29] Ward E., Halpern M., Schrag N. (2008). Association of insurance with cancer care utilization and outcomes. *CA Cancer Journal for Clinicians*.

